# Self-monitoring blood pressure in pregnancy: evaluation of women’s experiences of the BUMP trials

**DOI:** 10.1186/s12884-024-06972-4

**Published:** 2024-11-28

**Authors:** Alison Chisholm, Katherine L Tucker, Carole Crawford, Marcus Green, Sheila Greenfield, James Hodgkinson, Layla Lavallee, Paul Leeson, Lucy Mackillop, Christine McCourt, Jane Sandall, Hannah Wilson, Lucy C Chappell, Richard J McManus, Lisa Hinton

**Affiliations:** 1 Nuffield Department of Primary Care Health Sciences, University of Oxford, Oxford, UK; 2 Action on Pre-eclampsia, The Stables, 80 B High Street, Evesham, Worcestershire UK; 3 Institute of Applied Health Research, University of Birmingham, Birmingham, UK; 4 Cardiovascular Clinical Research Facility, Division of Cardiovascular Medicine, University of Oxford, Oxford, UK; 5 Nuffield Department of Women’s & Reproductive Health, University of Oxford, Oxford, UK; 6 Centre for Maternal & Child Health Research, School of Health Sciences, City, University of London, London, UK; 7 Department of Women and Children’s Health, King’s College London, St Thomas’ Hospital, London, UK

**Keywords:** Blood pressure, Self-monitoring, Pregnancy, Hypertension, Pre-eclampsia, Remote care, Process evaluation

## Abstract

**Background:**

The COVID-19 pandemic accelerated the adoption of remote care, or telemedicine, in many clinical areas including maternity care. One component of remote care, the use of self-monitoring of blood pressure in pregnancy, could form a key component in post-pandemic care pathways. The BUMP trials evaluated a self-monitoring of blood pressure intervention in addition to usual care, testing whether it improved detection or control of hypertension for pregnant people at risk of hypertension or with hypertension during pregnancy. This paper reports the qualitative evaluation which aimed to understand how the intervention worked, the perspectives of participants in the trials, and, crucially, those who declined to participate.

**Methods:**

The BUMP trials were conducted between November 2018 and May 2020. Thirty-nine in-depth qualitative interviews were carried out with a diverse sample of pregnant women invited to participate in the BUMP trials across five maternity units in England.

**Results:**

Self-monitoring of blood pressure in the BUMP trials was reassuring, acceptable, and convenient and sometimes alerted women to raised BP. While empowering, taking a series of self-monitored readings also introduced uncertainty and new responsibility. Some declined to participate due to a range of concerns. In the intervention arm, the performance of the BUMP intervention may have been impacted by women’s selective or delayed reporting of raised readings and repeated testing in pursuit of normal BP readings. In the usual care arm, more women were already self-monitoring their blood pressure than expected.

**Conclusions:**

The BUMP trials did not find that among pregnant individuals at higher risk of preeclampsia, blood pressure self-monitoring with telemonitoring led to significantly earlier clinic-based detection of hypertension nor improved management of blood pressure. The findings from this study help us understand the role that self-monitoring of blood pressure can play in maternity care pathways. As maternity services consider the balance between face-to-face and remote consultations in the aftermath of the COVID-19 pandemic, these findings contribute to the evidence base needed to identify optimal, effective, and equitable approaches to self-monitoring of blood pressure.

## Background

Raised blood pressure (BP) affects 10% of pregnant women worldwide [1], is a leading cause of maternal mortality and morbidity and a factor in 14% of maternal deaths and 15% of stillbirths globally [2, 3].

BP monitoring is a key element of antenatal care. Self-monitoring of BP involves BP readings taken by individuals outside clinical settings and has been shown to support the detection and management of hypertension in the general population [4–6]. A self-monitoring intervention with telemonitoring was developed and piloted for use in pregnancy [7–9]. Self-monitoring of BP in pregnancy allows more frequent readings with the potential to detect hypertension between antenatal appointments and to obtain readings in varied circumstances, providing an expanded view of BP over time and indicating where clinic readings diverge from self-monitored readings. Self-monitoring of BP could reduce additional clinic visits and can involve pregnant people more closely in their care [9, 10]. It also has the potential to entail responsibilisation, whereby individuals become responsible for a task previously undertaken by healthcare professionals [11–13]. A self-monitoring intervention with telemonitoring was developed, using the Person-based approach, and piloted for use in pregnancy [14].

The self-monitoring of BP intervention was evaluated in the BUMP1 and BUMP2 trials. The BUMP1 trial tested whether self-monitoring of BP improved detection of hypertension in normotensive women at higher risk of developing hypertension, alongside usual care; the BUMP2 trial aimed to examine its effectiveness in controlling BP in individuals with pregnancy hypertension. The self-monitoring intervention resulted in no earlier clinic detection of hypertension in BUMP1 nor improved management of BP in BUMP2 [15–17].

This article reports findings from the BUMP1 and BUMP2 trials’ qualitative process evaluation. It aimed to understand participants’ experiences of self-monitoring of BP in the BUMP trials, how the intervention worked and exerted its effects and to give further context to the trial outcomes. The evaluation also explored perspectives of individuals who declined to take part in the trial and those in the usual care arm of the BUMP2 trial to find out whether they self-monitored their BP.

## Methods

**Fig. 1:**
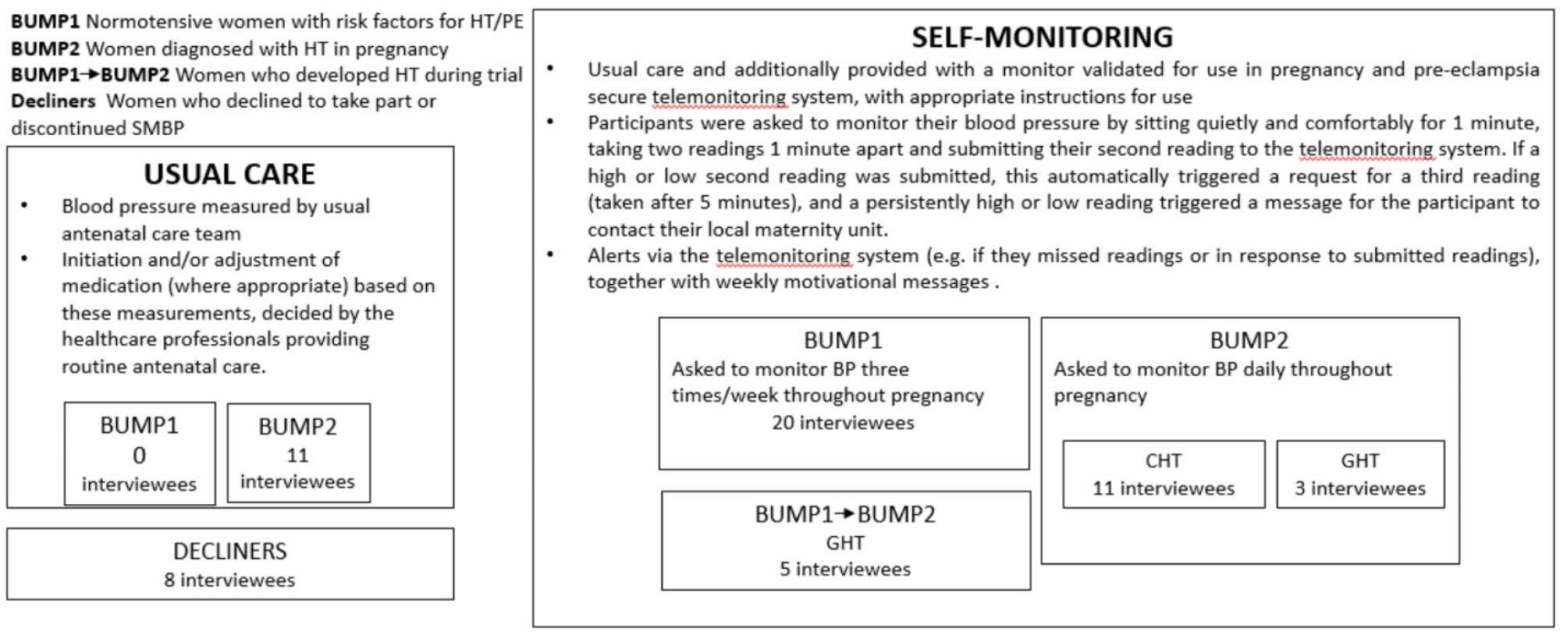
Trial and interview participants

Five trial sites in England were purposively selected to sample participants for the evaluation interviews, including maternity units from large teaching hospitals and smaller non-teaching hospitals, geographical spread, ethnic and socioeconomic diversity of populations served. Purposive sampling was used to achieve a maximum diversity sample in terms of socioeconomic status (using highest educational attainment as a proxy); ethnic background and parity. The sample size was designed to achieve information power [18]. In-depth interviews were carried out between July 2018 and October 2019 (see Fig. 1). Brief telephone interviews were conducted retrospectively with 11 people randomised to the usual care arm of the BUMP2 trial at two sites.

Trial participants and women who declined participation were invited to take part by research midwives during trial recruitment. Written informed consent was obtained by researcher (AC).

Interviews were carried out face-to-face or by telephone, according to preference. Face-to-face interviews took place either in a healthcare setting, after an antenatal consultation, at the participant’s home, or occasionally in a public place.

The interview guide was based on a review of the existing literature on self-monitoring of BP in pregnancy, and the trial’s development work. Interviews were undertaken by an experienced social science researcher. Interviews lasted between 30 and 60 min, apart from the usual care interviews which were shorter, lasting between 15 and 30 min. Interviews were recorded and transcribed verbatim. A coding frame was developed based on the research aims and incorporating additional themes that emerged from the data. Themes were developed by social scientists AC and LH, and discussed with the wider research team that included clinicians and maternity care researchers. Interviews were coded in NVivo 12 and inductive and deductive thematic analysis carried out [19]. Analysis was led by AC and LH, with regular input from the wider study team. Ethical approval was gained from the West Midlands - South Birmingham NHS Research Ethics Committee: ref 17/WM/0241.

## Results

### Sample

We interviewed a total of 58 women (see Table 1). Thirty-nine trial participants (out of 3291) randomised to the intervention arm of the trials were interviewed. Twenty of these had been recruited to the BUMP1 trial. Fourteen had been recruited to the BUMP2 trial, of whom 11 had chronic hypertension and three had gestational hypertension. After the trial finished recruitment, a further five participants were initially recruited to BUMP1 before migrating to BUMP2, as per trial protocol, when they submitted raised readings [15–17]. We also interviewed eight pregnant people who declined or discontinued their trial participation. After the trial finished recruitment, a further eleven BUMP2 participants who had been randomised to the usual care arm of the trial were interviewed by phone (January – March 2021). People from minority ethnic and lower socioeconomic groups were well represented in the sample. (Table 1). The educational attainment of participants was somewhat higher than in the population as a whole, but was a closer match to the population than is often the case in research studies. We were able to access full demographic data for all the women currently enrolled in the trial (*n* = 39) but not for the 8 who ‘declined’ or the women in the ‘usual care’ arm who were interviewed by phone in 2021.

**Table 1:** Ethnicity, educational achievement, and parity of interview participants

	BuMP1 (No HT)	BuMP2 (CHT)	BuMP2 (GHT)	BuMP1-2 (GHT)	Declined	BuMP2 usual care	Row Total
**Ethnicity**							
Asian or Asian British	1	0	0	1	0	1	**3**
Black or Black British	1	5	0	0	0	3	**9**
Mixed	2	0	1	0	0	0	**3**
White British	14	3	2	2	5	4	**30**
White other	2	3	0	2	0	3	**10**
Unassigned	0	0	0	0	3	0	**3**
**Highest educational achievement**							
No formal qualifications	0	0	0	0	0	1	**1**
GCSE/O-level equivalent	0	0	0	0	0	0	**0**
Vocational qualifications	0	2	0	0	0	0	**2**
A-Level	4	2	0	0	0	1	**7**
Professional qualifications	0	3	0	0	0	2	**5**
First Degree	7	2	1	1	2	5	**18**
PG or equivalent	9		2	4	1	1	**17**
Unassigned	0	2	0	0	5	1	**8**
**Parity**							
0	7	5	1	3	5		**21**
1	10	3	2	2	1		**18**
2	3	1	0	0	2		**6**
3	0	0	0	0	0		**0**
4+	0	2	0	0	0		**2**
Unassigned	0	0	0	0	0	11	**11**
**Column total**	**20**	**11**	**3**	**5**	**8**	**11**	**58**

Supporting quotes are presented, each followed by the participant’s ID number; whether they took part in the BUMP1 or BUMP2 trial, or moved from BUMP1 to BUMP2; their parity, highest educational achievement, and ethnic group, where available.

In the following sections we present the five broad themes that emerged from the analysis. These explore :

- the acceptability and burden of self-monitoring;
- the convenience of self-monitoring and reflections on the reassurance, and confidence that self-monitoring offered;
- participants’ reflections on how self-monitoring impacted on empowerment, advocacy and informed-decision making;
- clinical engagement;
- the ambiguity and uncertainty associated with self-monitoring.

### Acceptability and burden

Women’s accounts of self-monitoring of BP were positive, consistent with pilot findings [9]. Most reported self-monitoring their BP was quick, easy and could be incorporated into daily life. Interviews suggested overall acceptability, low burden, and high self-efficacy; significantly participants felt confident they were able to carry out self-monitoring.

Those who understood their risk of developing raised BP saw self-monitoring as an easy way to mitigate the risk that elevated BP might go undetected. Interviews suggested self-monitoring of BP was common among the pregnant people with hypertension in the trial’s usual care arm. However, a small number, in the intervention group, who perceived their risk of hypertension to be very low, felt the burden of self-monitoring outweighed the likely benefit, but participated in the trial nonetheless.

Reasons for declining trial participation were varied. Some declined due to fears they might become preoccupied with monitoring. Others were concerned self-monitoring of BP would exacerbate their anxiety.

*Just trying to think*,* I think maybe doing it too much in one day*,* like*,* if you’re just constantly taking readings. I think the dietician might have said to me*,* you know*,* like*,* “You don’t need to take your blood pressure all the time. You don’t need to do it four times a day.” I think she wanted to just make sure I wasn’t becoming obsessive with the*,* with the machine which can sometimes happen with people.* (Declined, 3)

A woman with previous experience of pre-eclampsia declined to take part in the trial in order to protect herself from anxiety. She had come off antidepressant medication due to her pregnancy and was working hard to manage her anxiety and maintain good mental health; she saw self-monitoring her BP as a potential threat to that.

*I know the days that I would have to take my blood pressure*,* it would be on my mind all day and I’d wind myself up to point that I had high blood pressure… I’m going to have to work really hard to remain positive and keep myself grounded that everything’s going to be okay and I would have had to have opted out of the study then anyway because my head would have been all over the place. … Yes*,* it just isn’t for me because of how*,* how my mind works.* (Declined, 5)

Others declined because they perceived their risk of hypertension was low and were insufficiently motivated to monitor their BP.

Interviews with pregnant people with a diagnosis of hypertension (BUMP2 participants) in the usual care arm of the trial revealed that a proportion of them independently self-monitored their BP during their trial participation, often daily or several times a week, finding it easy and a valuable indicator of rising BP. This independent self-monitoring potentially diluted the trial’s effects.

*It also gave me peace of mind… at one point in this pregnancy I did get admitted with high blood pressure and actually that was because I didn’t feel very well and I went and test*,* you know*,* in this routine testing it was really high and I tried that a few times willing it to go down and it wouldn’t. So*,* I went off to the midwife and I was admitted so*,* so in that respect it helped me potentially*,* you know*,* avoid an issue because I realised my blood pressure was high. Otherwise*,* I would never have known….* (Usual care, 1)

### Convenience, reassurance and confidence

Self-monitoring of BP allowed participants to obtain more frequent BP readings. The convenience of self-monitoring of BP was valued particularly by those who had work or caring responsibilities and for whom making a clinic appointment might entail travelling, lengthy waiting times and additional stress that may have led to raised readings. Self-monitoring allowed women to avoid making additional, non-routine clinic visits simply to check their BP if feeling unwell.

*Yes*,* it was convenient because I could do it in my own time*,* sitting in my pyjamas*,* watching the telly and didn’t have to worry about booking an appointment with the surgery or calling the midwife. I know they’re all busy and they’ve got hundreds of ladies to look after*,* so I’m not the only one. So*,* I could do it at my own pace and my own environment which is helpful as well because you’re sort of more relaxed and your readings may be more accurate than queuing in surgery*,* waiting for an appointment.* (21: BuMP1-2, P1, PG degree, White Other)

*If I were to have a headache*,* a bad headache*,* I’d probably start thinking about my blood pressure. I think when you’re feeling ill with a migraine*,* you don’t want to be going to a midwife… if I can quickly check my blood pressure at home then excellent*,* that just puts your mind at rest for that as well.* (35: BuMP1, P2, PG degree, White British)

Women reported self-monitored BP readings had a role in reducing perceived stress or anxiety. Normal self-monitored readings provided reassurance, giving participants confidence their BP was not creeping up undetected (although they were given information emphasising they should not be reassured by normal BP if they had other relevant symptoms). For those whose clinic BP readings were systematically higher than their self-monitored readings (white coat hypertension, or WCH), self-monitoring mitigated anxiety associated with clinic visits [20]. Obtaining repeated normal self-monitored readings reduced their anxiety that clinic readings would be high, which in some cases led to a reduction in the discrepancy between self-monitored and clinic readings, reducing the extent of WCH.

*It’s quite interesting the home reading’s starting to match up with when I’m coming in… so at the beginning there was an imbalance and now the last couple of appointments have been quite similar… It used to be*,* ‘Oh it’s going to be high and they what are they going to do?’ Whereas I just don’t stress about it too much quite honestly. Yes*,* it definitely does make a difference.* (19: BuMP2, P1, education level unknown, White British)

### Empowerment, advocacy, and informed decision-making

Self-monitoring reduced participants’ reliance on clinicians for monitoring their BP between routine antenatal visits and promoted feelings of independence and empowerment. Some reported self-monitoring of BP had alerted them to raised BP that would otherwise have gone unnoticed until a routine antenatal appointment. One woman, who developed gestational hypertension that was first detected by her self-monitoring, felt monitoring her own BP allowed her to “take charge” of her own medical care.

*R: I don’t think I would have got to that hypertension clinic if it hadn’t have been for taking my blood pressure at home… I just wouldn’t have known that my blood pressure had got that high … Yeah and otherwise …I would have just stuck to my routine antenatal care*.

I: So you think it was spotted earlier because of the trial?

*R: Oh absolutely…. The BUMP study I’m very grateful for because I see it as like the beginning of actually*,* I don’t know whether it’s like taking charge of my own medical care in a way*,* because otherwise the doctors would have said no more scans*,* I wouldn’t have had the blood pressure tablets. I would have been at home not knowing what my blood pressure was because I had no symptoms…I had extreme swelling*,* that’s all I had*,* but pregnancy swelling is normal*,* so you just think*,* ‘Ah I’m just a pregnant woman who’s swollen. I’ll just do what the doctors say.’ (9: BuMP1-2 (GHT)*,* P0*,* PG*,* White British)*.

Self-monitoring often increased women’s embodied knowledge and familiarity with their BP, its variability, and the factors that raised or lowered it. It could foster confidence in interpreting their readings. By developing an understanding of the influence on their BP of circumstances or their emotional or psychological state (for example, timing of medication, sleep, work or family stressors), they could target these factors to bring it closer to a “normal” level.

*I’ve got a little boy and if I’m running around after him or doing something*,* it definitely does affect it quite*,* quite a lot actually*,* like*,* it jumps quite a bit*,* so I do have to sit for a bit and kind of rest. So the mornings aren’t always the best trying to get out. Usually the evenings is a little bit easier.* (19: BuMP2, P1, Education unknown, White British)

Regular self-monitored readings allowed pregnant people to obtain clinically relevant information otherwise unavailable to them and their clinicians. A participant described how understanding her own “normal” home BP patterns allowed her to identify anomalous readings and request further clinic measurements.

*I suppose what the study allows you to do is have ways round what you feel is kind of like your regular blood pressure*,* so when you do come into hospital and they do take a reading and if it does look slightly odd*,* you could always ask them to take a second one…. because you know that’s quite outside of what you usually monitor and from your own monitoring what you get.* (12: BuMP1, P1, First Degree, Black or Black British)

Another said self-monitored readings enabled her to identify BP readings that, while still within normal range, were elevated relative to her own “normal.” This helped her to predict and to prepare psychologically for the subsequent development of hypertension and pre-eclampsia.

Information held in series of self-monitored readings recorded on the app gave women confidence to advocate for the care they wanted and provided evidence that reassured them they had legitimate cause to seek help.

*“…because you can’t just turn up at the practice and say I want my blood pressure done now. Whereas this way*,* you can do it yourself and then you*,* you ring and say*,* “It’s higher. I need to someone*,* to see someone*,* sort of*,* urgently.”” _(11: BUMP1*,* P1*,* Postgraduate qualifications; White British)*.

Where healthcare professionals regarded self-monitored readings as legitimate and relevant to the clinical picture, they were incorporated into decision-making. In some cases, these decisions were made collaboratively between pregnant people and healthcare professionals. But this was not uniform. Occasionally participants gave accounts of uncomfortable, tense encounters, where self-monitored readings were not trusted by healthcare professionals, who felt medication or hospital admission was appropriate but the woman felt her normal self-monitored readings meant this was unnecessary.

*She kind of did dismiss them*,* like*,* “Oh but you’re measuring at home.”… It was really quite bizarre… But I guess at least it strengthened the confidence that I was doing the right thing and there really wasn’t anything wrong and as long as I was feeling fine*,* I had the proof that my body was doing what it should be doing. (24: BuMP2 (WCH)*,* P1*,* PG degree*,* Mixed)*

### Clinician engagement

Women’s experiences of their healthcare professionals’ willingness to engage with their self-monitored readings ranged widely, from full interest and openness to disinterest or distrust, and depended on whether the woman’s BP was perceived as a risk. BUMP1 participants (those with risk factors), particularly those whose clinic readings had always been normal, commonly reported they had not been asked about their self-monitored readings by healthcare professionals.

*R: They’re only interested in my reading when I’m at the doctors.* (25: BuMP1, P1, First degree)

BUMP2 participants (those with hypertension) were more often asked about their self-monitored readings. Some obstetricians actively encouraged BUMP2 participants to share self-monitored readings and took them into account in determining treatment. Where a pregnant person had high BP readings or a history of pre-eclampsia, obstetricians and midwives usually appeared to take a greater interest in self-monitored readings.

*Yeah*,* they’re interested in the reading which helps them to*,* to be confident in what they are doing or*,* because they don’t want what has happened previously to happen.* (14: BuMP2 (CHT), P2, Prof Quals, Black or Black British)

Participants in the usual care arm also discussed their home readings with healthcare professionals; in some cases, these influenced care decisions. However, not all health professionals engaged with these self-monitored readings.

*I was surprised because … especially on a graph you can see over the months how*,* how it’s changing and you see*,* like*,* the high points. Like when it was really high and you can even really scroll down and look at even the timings if it’s*,* you know*,* being measured in the morning*,* evening*,* how*,* you know*,* if I was travelling or where I was. I was surprised because it should be as a source of data and could help.* (14: BuMP2 (CHT), P2, Prof Quals, Black or Black British)

### Ambiguity and uncertainty

Participants’ increased understanding of factors influencing their BP and its fluctuations led to new judgements and responsibilities; whether and when to take readings; whether and when to act on raised readings (recording them in the app or contacting healthcare services). The intervention did not specify precisely the time to take BP readings (the pilot work suggested flexibility was important [9]) or provide a facility to record caveats/explanations for occasions when they felt her self-monitored reading did not represent her regular BP.

*Yeah because… there isn’t like a comment*,* so I couldn’t put ‘I didn’t sit down for five minutes*,*’ or ‘I’ve had salt and vinegar crisps.’ or*,* you know*,* whatever it is. But then that wouldn’t be necessary for everyone*,* it’s only because I’m aware of things that might trigger it*,* so.* (18: BuMP1, P1, First Degree, White British)

Uncertainty about the significance of a raised reading, attributable to an identifiable factor, sometimes led to selective or delayed reporting or acting on higher readings, particularly if the circumstances made it difficult or inconvenient to attend clinic.

Participants described personal judgments that impacted on whether they acted on a raised reading. A BUMP2 participant said they would monitor again the following morning if they recorded a high reading, and if it was still high, take BP medication before taking another reading.

I: Do you always put in the reading?

*R: Yeah*,* I will put*,* I will put it on and then it will ask me to monitor it again. If I monitor it again*,* it shows high reading*,* I will just ignore it. The reading is high again*,* I will just ignore it*,* leave the first one and then in the morning*,* I will do it again. If it is high*,* I will leave*,* take my medication and then do it again*,* then put whatever the outcome is.* (14: BuMP2, P2 Professional qualifications, Black or Black British)

A BUMP1 participant said they could imagine obtaining a higher reading and feeling ambivalent about entering it. She had a stressful, tiring job with a long commute, and a lot of responsibility, which would make her reluctant to take time off for a clinic appointment or to manage her BP. If she identified stress as a factor, she would monitor again when she felt calmer to avoid the inconvenience of going to clinic.

*R: Yes*,* because that would probably be my first tactic to see if it did come down and it would also I guess depend on what I’d just been doing… or if I know I’m worried about something or if there were any other factors that might be influencing it*,* I might try and rule those out first before I was thinking*,* ‘Okay*,* yeah. I’ve definitely got high blood pressure.’* (33: BuMP1, P0, First Degree, White British).

Others described attributing raised readings to external factors - being too busy, a hot bath or stressful meeting - and would consider them unrepresentative and not record them.

*… maybe if I’ve had a particular salty dinner*,* I might avoid it that night*,* but no*,* there’s nothing really that puts me off. It’s just…. I guess there’s an element of playing the system that if I know it might be high*,* I might have to do more.* (18: BuMP1, P1, First Degree, White British)

*… you could convince yourself that this wasn’t representative of today and so I’m not going to enter it because*,* yeah I don’t know*,* I just had a massively stressful meeting.* (50: BuMP2 (WCH/GHT), P0, Postgraduate Qualifications, White British)

Some described efforts to obtain a “normal” BP reading, for example by rehearsing calming messages to achieve a state of relaxation. Some avoided taking a reading at all on a day when they anticipated that their BP might be higher than usual. Through regular self-monitoring, many participants recognised daily rhythms of BP fluctuations. They chose to monitor their BP consistently at the time of day they considered their BP to be “representative”, at its lowest, or to avoid “spikes”.

*It took me a little while to kind of figure out what was working best for me …I found the morning worked best for me basically*,* first thing in the morning*,* get out of bed and take it.* (42: BuMP1, P0, Postgraduate Qualifications, White British)

For some, attributing meaning to a higher reading could lead to a decision not to act, but instead to repeat readings until they got a reading they “liked”. One participant, aware of the potential danger of raised BP having previously experienced serious complications with pre-eclampsia, discontinued self-monitoring as they were unable to obtain a normal reading and found the effort to do so stressful.

## Discussion

As health systems recover from the COVID-19 pandemic and reflect on the future role of telemedicine in maternity services, the findings from this study of women’s experiences of self-monitoring of BP in pregnancy are valuable. For most participants, self-monitoring of BP was reassuring, acceptable and convenient. Women reported self-monitoring improved their embodied knowledge, introducing awareness of normal daily fluctuations in BP and an understanding of the significance of contextual factors. This arguably gave women a deeper knowledge of their BP than their healthcare professionals and allowed them to advocate for the care they wanted. Self-monitored readings alerted some to rising BP. Those who preferred not to self-monitor cited concerns it would increase their anxiety, fear of becoming preoccupied, low perceived risk of hypertension, or preferring to have a healthcare professional present. In the context of the BUMP trials, self-monitoring of BP was additive, allowing participants to share otherwise unavailable information that potentially contributed to decisions about their care. Whether women found self-monitoring of BP empowering depended to some extent on the response from their healthcare professionals, how seriously they considered self-monitored readings, and the quality of those interactions. Good communication between the woman and healthcare professional allowed informed decisions to be made on a shared understanding of BP.

Self-monitoring of BP, and the ability to take readings under *any* circumstances, shifted women’s responsibilities in pregnancy. It introduced a range of new tasks for women and required them to make judgments that included interpreting and managing ambiguity around whether to act on a raised reading. In the trial participants received instructions on what to do if their BP was high, but the judgements about whether and when to take readings, or to act on raised readings, rested with them. Some delayed reporting and acting on raised readings, reported readings selectively or took multiple readings in pursuit of normal readings. Inviting individuals to become responsible for a task previously undertaken by healthcare professionals and to manage the ambiguities associated with fluctuating self-monitored readings, or discrepancies between self-monitored and clinic readings, is part of a general shift towards responsibilisation [11–13]. Emerging evidence shows while maternity service users and health professionals value the convenience and flexibility of remote care, of which self-monitoring is one component, it is important to recognise there are both advantages and disadvantages, not least in regard to inequalities and these new responsibilities [21, 22].

### Strengths and limitations

This evaluation drew on in-depth interviews with a large, diverse sample of participants in the self-monitoring arm of the BUMP trials, including those from deprived and minority ethnic backgrounds (at higher risk of hypertension in pregnancy and poorer outcomes), participants in the usual care arm of the trials and others who declined participation. Existing evidence on self-monitoring interventions in pregnancy has not robustly captured the perspectives of women of ethnic minority backgrounds, refugees, people experiencing homelessness, people with poor fluency in English; the enduring poor maternal outcomes for these groups makes including their voices critical [23, 24]. These data are therefore a valuable contribution to the evidence base, although we acknowledge we were not able to capture the perspectives of refugees and those with poor fluency in English.

This was a pragmatic study with self-monitoring of BP used in addition to usual care [15] which completed recruitment before the Covid-19 pandemic. Different results might be found if self-monitoring of BP were combined with health system change and modified care pathways rather than these factors being left to individual clinicians. A larger sample of individuals from the usual care arm would have allowed greater insight into the likely effects of their self-monitoring of BP on the trial outcomes.

### Interpretation

The BUMP trials established that self-monitoring of BP in pregnancy in addition to usual care is safe, although the intervention was not found to be effective overall in achieving earlier clinic detection of hypertension in BUMP1 or improved management of BP in BUMP2 [16, 17]. Interviews with participants in the usual care arm reflect the pre-pandemic survey findings that 50% of pregnant women with hypertension monitor their BP at home, although few share their self-monitored readings with their healthcare professionals [25]. We hypothesise that high levels of self-monitoring of BP in the general population and in the usual care arm may have contributed to similarities in outcomes between the randomised groups [25]. The insights of this evaluation are therefore important as we think about the design of future maternity services, in particular as women are taking on this responsibility already. The interview data reveal that individuals sometimes delayed acting on raised BP readings, which may partly explain why the trials revealed no improvement in detection times. The selective or delayed reporting of raised readings resonates with other research that pregnant people sometimes delayed help-seeking where it conflicts with other family commitments [26]. This, along with multiple readings taken in pursuit of normal values could have affected the intervention’s performance with respect to primary outcome measures. Implementation of self-monitoring of BP into antenatal care pathways must ensure women are given clear information about the importance of reporting raised readings in a timely manner and clear instructions on escalation pathways. Self-monitoring of BP should be considered additive, and the existing schedules of antenatal consultations with women maintained.

In many respects, experiences of self-monitoring of BP in pregnancy were found to be similar to those of the non-pregnant population [27]. In line with previous research with type 2 diabetes patients [28] and hypertensive patients [29], this study found women valued having more frequent home readings, believing these provided a rounder picture of their BP than clinic readings only, in a more convenient setting. For most, it allowed them to develop embodied knowledge [30] and a “feel” for their BP and how it varied in different circumstances [31]. In some cases, self-monitored readings facilitated patient-clinician interactions in consultations about hypertension and bridged a potential gap in the traditional relationship between the two parties [32]. The use of the readings to provide a full clinical picture and collaborative decision-making and care planning between clinician and the woman could be valuable to future care pathway design and support ambitions towards patient-centred care [33].

The perceived reluctance of some healthcare professionals to incorporate self-monitored readings into decision-making in pregnancy (particularly where self-monitored readings are normal and clinic readings are elevated) may reflect temporal concerns about how recent self-monitored readings were. They may also reflect concerns about the acute and sudden risk to the health of pregnant people and their babies presented by elevated BP and pre-eclampsia, in contrast to the more chronic effects of raised BP outside of pregnancy [34, 35]. The present study suggests that more information about how to interpret self-monitoring of BP readings would be valuable for women and healthcare professionals [32]. 

Post-trial, self-monitoring of BP in pregnancy was implemented during the Covid19 pandemic in many settings, alongside a rapid shift to remote antenatal care provision, in response to recommendations from the Royal College of Obstetricians and Gynaecologists to reduce face-to-face consultations [36, 37]. An English NHS initiative gave maternity units monitors to distribute to pregnant people with hypertension or at risk of pre-eclampsia [38, 39] and a Scottish NHS initiative distributed 5000 BP monitors to 14 NHS health boards to give to enable women to undertake supported remote self-monitoring of their BP [40]. Evaluations of these initiatives confirmed that women found self-monitoring easy to use and reassuring, and they valued the convenience it offered. However, analysis of the impact of remote antenatal care more broadly cautions that the implementation of major changes to healthcare systems are rarely straightforward, and that new models of care should be sensitised to equity and inclusion and play close attention to issues of access and any unintended consequences of increased responsibilisation [21]. 

## Conclusion

In an era when telemedicine is promoted by policy-makers [41, 42], and substantial proportions of the pregnant population are already self-monitoring, further research is needed to explore the potential for self-monitored BP readings to contribute to informed decision-making in pregnancy. This must pay close attention to the health equity, any potential unintended consequences of increased responsibilisation and include developing clearer counselling to support pregnant people in their judgements about whether to act on a raised reading and guidance to help them and healthcare professionals to interpret variation in readings and manage discrepancies between self-monitored and clinic readings.

## Data Availability

The datasets generated and/or analysed during the current study are not publicly available due to them containing information that could compromise research participant privacy/consent, but are available from the corresponding author on reasonable request.

## References

[1] Abalos E, Cuesta C, Grosso AL, Chou D, Say L. Global and regional estimates of preeclampsia and eclampsia: a systematic review. Eur J Obstet Gynecol Reprod Biol. 2013;170(1):1–7. 23746796 10.1016/j.ejogrb.2013.05.005

[2] Lawn JE, Blencowe H, Waiswa P, Amouzou A, Mathers C, Hogan D, et al. Stillbirths: rates, risk factors, and acceleration towards 2030. Lancet. 2016;387(10018):587–603. 26794078 10.1016/S0140-6736(15)00837-5

[3] Say L, Chou D, Gemmill A, Tunçalp Ö, Moller AB, Daniels J, et al. Global causes of maternal death: a WHO systematic analysis. Lancet Glob Health. 2014;2(6):e323–33. 25103301 10.1016/S2214-109X(14)70227-X

[4] Baral-Grant S, Haque MS, Nouwen A, Greenfield SM, McManus RJ. Self-monitoring of blood pressure in hypertension: a UK Primary Care Survey. Int J Hypertens. 2012;2012:582068. 22013510 10.1155/2012/582068 PMC3195273

[5] Constanti M, Boffa R, Floyd CN, Wierzbicki AS, McManus RJ, Glover M. Options for the diagnosis of high blood pressure in primary care: a systematic review and economic model. J Hum Hypertens. 2021;35(5):455–61. 32461579 10.1038/s41371-020-0357-x PMC8134050

[6] Tucker KL, Sheppard JP, Stevens R, Bosworth HB, Bove A, Bray EP, et al. Self-monitoring of blood pressure in hypertension: a systematic review and individual patient data meta-analysis. PLoS Med. 2017;14(9):e1002389. 28926573 10.1371/journal.pmed.1002389 PMC5604965

[7] Tucker KL, Taylor KS, Crawford C, Hodgkinson JA, Bankhead C, Carver T, et al. Blood pressure self-monitoring in pregnancy: examining feasibility in a prospective cohort study. BMC Pregnancy Childbirth. 2017;17(1):442. 29284456 10.1186/s12884-017-1605-0 PMC5745883

[8] Band R, Hinton L, Tucker KL, Chappell LC, Crawford C, Franssen M, et al. Intervention planning and modification of the BUMP intervention: a digital intervention for the early detection of raised blood pressure in pregnancy. Pilot Feasibility Stud. 2019;5(1):153. 31890265 10.1186/s40814-019-0537-z PMC6925434

[9] Hinton L, Tucker KL, Greenfield SM, Hodgkinson JA, Mackillop L, McCourt C, et al. Blood pressure self-monitoring in pregnancy (BuMP) feasibility study; a qualitative analysis of women’s experiences of self-monitoring. BMC Pregnancy Childbirth. 2017;17(1):427. 29258469 10.1186/s12884-017-1592-1 PMC5735874

[10] Hodgkinson JA, Tucker KL, Crawford C, Greenfield SM, Heneghan C, Hinton L, et al. Is self monitoring of blood pressure in pregnancy safe and effective? BMJ. 2014;349:g6616. 25406132 10.1136/bmj.g6616

[11] Varey S, Dixon M, Hernández A, Mateus C, Palmer TM, Milligan C. The role of combinatorial health technologies in supporting older people with long-term conditions: responsibilisation or co-management of healthcare? Soc Sci Med. 2021;269:113545. 33339684 10.1016/j.socscimed.2020.113545

[12] Hinton L, Chisholm A, Jakubowski B, Greenfield S, Tucker KL, McManus RJ, et al. You probably won’t notice any symptoms: blood pressure in pregnancy—discourses of contested expertise in an era of self-care and responsibilization. Qual Health Res. 2021;31(9):1632–44. 34116606 10.1177/10497323211003067 PMC8438769

[13] Davies B. ‘Personal Health Surveillance’: the Use of mHealth in Healthcare Responsibilisation. Public Health Ethics. 10.1093/phe/phab013 PMC8661076 34899983

[14] Band R, Hinton L, Tucker KL, Chappell LC, Crawford C, Franssen M, et al. Intervention planning and modification of the BUMP intervention: a digital intervention for the early detection of raised blood pressure in pregnancy. Pilot Feasibility Stud. 2019;5(1):1–12. 31890265 10.1186/s40814-019-0537-z PMC6925434

[15] Dougall G, Franssen M, Tucker KL, Yu L-M, Hinton L, Rivero-Arias O, et al. Blood pressure monitoring in high-risk pregnancy to improve the detection and monitoring of hypertension (the BUMP 1 and 2 trials): protocol for two linked randomised controlled trials. BMJ Open. 2020;10(1):e034593. 31980512 10.1136/bmjopen-2019-034593 PMC7044851

[16] Chappell L, Tucker KL, Galal U, Yu L, Campbell H, Rivero-Arias O, Allen J, Band R, Chisholm A, Crawford C, Dougall G, Engonidou L, Franssen M, Green M, Greenfield S, Hinton L, Hodgkinson J, Lavallee L, Leeson P, McCourt C, Mackillop L, Sandall J, Santos M, Tarassenko L, Velardo C, Wilson H, Yardley L, McManus RJ. Effect of self-monitoring of blood pressure on blood pressure control in pregnant individuals with chronic or gestational hypertension: the BUMP 2 randomized trial. In press.

[17] Katherine L, Tucker SM, Yu L-M, Campbell H, Rivero-Arias O, Wilson HM, Allen J, Band R, Chisholm A, Crawford C, Dougall G, Engonidou L, Franssen M. Marcus Green, Greenfield, Lisa Hinton, James Hodgkinson, Layla Lavallee, Paul Leeson, Christine McCourt, Lucy Mackillop, Jane Sandall, Mauro Santos, Lionel Tarassenko, Carmelo Velardo, Lucy Yardley, Lucy C Chappell, and Richard J McManus. Effect of self-monitoring of blood pressure on diagnosis of hypertension during higher-risk pregnancy: the BUMP 1 randomized trial In press.

[18] Malterud K, Siersma VD, Guassora AD. Sample size in qualitative interview studies: guided by Information Power. Qual Health Res. 2016;26(13):1753–60. 26613970 10.1177/1049732315617444

[19] Braun V, Clarke V. Using thematic analysis in psychology. Qualitative Res Psychol. 2006;3:77–101.

[20] Tucker KL, Bankhead C, Hodgkinson J, Roberts N, Stevens R, Heneghan C, et al. How do home and clinic blood pressure readings compare in pregnancy? A systematic review and individual patient data meta-analysis. Hypertension. 2018;72(3):686–94. 30354754 10.1161/HYPERTENSIONAHA.118.10917 PMC6080884

[21] Hinton L, Dakin FH, Kuberska K, Boydell N, Willars J, Draycott T, et al. Quality framework for remote antenatal care: qualitative study with women, healthcare professionals and system-level stakeholders. BMJ Quality & Safety; 2022. 10.1136/bmjqs-2021-014329 PMC11041557 35552252

[22] Hinton L, Chisholm A, Jakubowski B, Greenfield S, Tucker KL, McManus RJ et al. You probably won’t notice any symptoms: blood pressure in pregnancy—discourses of contested expertise in an era of self-care and responsibilization. Qual Health Res. 2021:10497323211003067. 10.1177/10497323211003067 PMC8438769 34116606

[23] Hinton L, Kuberska K, Dakin F, Dixon-Woods M, Ekechi CBMJ. Opinion2021. https://blogs.bmj.com/bmj/2021/04/22/creating-equitable-remote-antenatal-care-the-importance-of-inclusion/

[24] Knight MBK, Patel R, Shakespeare J, Kotnis R, Kenyon S, Kurinczuk JJ, editors. On behalf of MBRRACE-UK. Saving lives, improving mothers’ Care Core Report - lessons learned to inform maternity care from the UK and Ireland Confidential enquiries into maternal deaths and morbidity 2018-20. Oxford: National Perinatal Epidemiology Unit; 2022.

[25] Tucker KL, Hodgkinson J, Wilson HM, Crawford C, Stevens R, Lay-Flurrie S, et al. Current prevalence of self-monitoring of blood pressure during pregnancy: the BUMP Survey. J Hypertens. 2021;39(5):994–1001. 33399304 10.1097/HJH.0000000000002734

[26] Carter W, Bick D, Mackintosh N, Sandall J. Maternal help seeking about early warning signs and symptoms of pre-eclampsia: a qualitative study of experiences of women and their families. Midwifery. 2021;98:102992. 33780789 10.1016/j.midw.2021.102992

[27] Tran K, Padwal R, Khan N, Wright M-D, Chan WS. Home blood pressure monitoring in the diagnosis and treatment of hypertension in pregnancy: a systematic review and meta-analysis. Can Med Association Open Access J. 2021;9(2):E642–50. 10.9778/cmajo.20200099 PMC8248564 34131027

[28] Hanley J, Fairbrother P, McCloughan L, Pagliari C, Paterson M, Pinnock H, et al. Qualitative study of telemonitoring of blood glucose and blood pressure in type 2 diabetes. BMJ Open. 2015;5(12):e008896. 26700275 10.1136/bmjopen-2015-008896 PMC4691739

[29] Jones MI, Greenfield SM, Bray EP, Baral-Grant S, Hobbs FD, Holder R, et al. Patients’ experiences of self-monitoring blood pressure and self-titration of medication: the TASMINH2 trial qualitative study. Br J Gen Pract. 2012;62(595):e135–42. 22520791 10.3399/bjgp12X625201 PMC3268493

[30] Clancy G, Boardman F, Rees S. Exploring trust in (bio)medical and experiential knowledge of birth: the perspectives of pregnant women, new mothers and maternity care providers. Midwifery. 2022;107:103272. 35151932 10.1016/j.midw.2022.103272

[31] Rickerby J, Woodward J. Patients’ experiences and opinions of home blood pressure measurement. J Hum Hypertens. 2003;17(7):495–503. 12821957 10.1038/sj.jhh.1001582

[32] Fletcher BR, Hinton L, Hartmann-Boyce J, Roberts NW, Bobrovitz N, McManus RJ. Self-monitoring blood pressure in hypertension, patient and provider perspectives: a systematic review and thematic synthesis. Patient Educ Couns. 2016;99(2):210–9. 26341941 10.1016/j.pec.2015.08.026

[33] Pilnick A. Reconsidering patient Centred Care: between autonomy and abandonment. Emerald; 2022.

[34] Flenady V, Wojcieszek AM, Middleton P, Ellwood D, Erwich JJ, Coory M, et al. Stillbirths: recall to action in high-income countries. Lancet. 2016;387(10019):691–702. 26794070 10.1016/S0140-6736(15)01020-X

[35] Knight M. The findings of the MBRRACE-UK confidential enquiry into maternal deaths and morbidity. Obstetrics. Gynecol Reproductive Med. 2019;29(1):21–3.

[36] Jardine J, Relph S, Magee LA, von Dadelszen P, Morris E, Ross-Davie M, et al. Maternity services in the UK during the coronavirus disease 2019 pandemic: a national survey of modifications to standard care. BJOG: Int J Obstet Gynecol. 2021;128(5):880–9. 10.1111/1471-0528.16547 32992408

[37] Royal College of Obstetricians and Gynaecologists. Coronavirus (COVID-19) infection and pregnancy. London, UK: Royal College of Obstetricians and Gynaecologists; 2021.

[38] Wilson H, Tucker K, Chisholm A, Hodgkinson J, Lavallee L, Mackillop L et al. Self-monitoring of blood pressure in pregnancy: a mixed methods evaluation of a national roll-out in the context of a pandemic. 10.1016/j.preghy.2022.07.006 PMC9364829 35933759

[39] Royal College of Obstetricians and Gynaecologists. Self-monitoring of blood pressure in pregnancy: information for healthcare professionals. London 2020.

[40] Paterson C, Jack E, McKinstry B, Whyte S, Denison FC, Cheyne H. Qualitative evaluation of rapid implementation of remote blood pressure self-monitoring in pregnancy during Covid-19. PLoS ONE. 2023;18(3):e0278156. 36862687 10.1371/journal.pone.0278156 PMC9980805

[41] NHS. NHS Long Term Plan. 2019.

[42] Department of Health and Social Care. Policy paper: Integration and innovation: working together to improve health and social care for all. London, UK: Her Majesty’s Stationery Office. 2021 January 2021.

